# Engineered Hsp70 chaperones prevent Aβ42-induced memory impairments in a *Drosophila* model of Alzheimer’s disease

**DOI:** 10.1038/s41598-018-28341-w

**Published:** 2018-07-02

**Authors:** Alfonso Martín-Peña, Diego E. Rincón-Limas, Pedro Fernandez-Fúnez

**Affiliations:** 1 Department of Neurology, McKnight Brain Institute, University of Florida, Gainesville, FL USA; 2 Center for Smell and Taste, University of Florida, Gainesville, FL USA; 3 Department of Neuroscience and Center for Translational Research on Neurodegenerative Diseases, Genetics Institute, University of Florida, Gainesville, FL USA; 4 Department of Biomedical Sciences, University of Minnesota Medical School, Duluth Campus, Duluth, MN USA

## Abstract

Proteinopathies constitute a group of diseases in which certain proteins are abnormally folded leading to aggregation and eventual cell failure. Most neurodegenerative diseases belong to protein misfolding disorders and, among them, Alzheimer’s disease (AD) is the most prevalent. AD is characterized by accumulation of the amyloid-β42 (Aβ42) peptide in the extracellular space. Hence, we genetically engineered a molecular chaperone that was selectively delivered to this cellular location. It has been reported that the heat shock protein 70 (Hsp70) binds Aβ42 preventing self-aggregation. Here, we employed two isoforms of the Hsp70, cytosolic and extracellular, to evaluate their potential protective effect against the memory decline triggered by extracellular deposition of Aβ42. Both Hsp70 isoforms significantly improved memory performance of flies expressing Aβ42, irrespective of their age or the level of Aβ42 load. Using olfactory classical conditioning, we established a *Drosophila* model of AD based on Aβ42 neurotoxicity and monitored memory decline through aging. The onset of the memory impairment observed was proportional to the cumulative level of Aβ42 in the *Drosophila* brain. These data support the use of this *Drosophila* model of AD to further investigate molecules with a protective activity against Aβ42-induced memory loss, contributing to the development of palliative therapies for AD.

## Introduction

Proteinopathies, also known as protein misfolding disorders, are neurodegenerative disorders characterized by an initial self-association of misfolded proteins that eventually aggregate into toxic assemblies. Chaperone activity of the heat shock protein 70 (Hsp70) has been reported to exert certain protective effects in some proteinopathies, including spinocerebellar ataxia (SCA), Parkinson’s disease (PD) and Alzheimer’s disease (AD)^1^. Early *in vitro* studies are consistent with a regulatory role of chaperones in protein misfolding and aggregation^2^. Together, these findings lead to the hypothesis that molecular chaperones are cellular machines modulating pathology through control of protein folding/misfolding.

Molecular chaperones are responsible for the proper folding and maturation of nascent proteins as well as the re-folding or degradation of misfolded ones. A large number of these chaperones are heat shock proteins (Hsps). These proteins were initially characterized for their response to heat shock stress, which is mostly regulated by gene expression via the heat shock factor 1 (Hsf1). Hsps trigger efficient responses against cellular stress^3^. Molecular chaperones can be found intracellularly (i.e.: Hsp40, Hsp60, Hsp70, Hsp90, Hsp100 and Hsp110) and extracellularly (i.e.: ST11, clustering and alpha-macroglobulin)^4^. In particular, the Hsp70 family of chaperones is known for its role in protein trafficking, folding of nascent proteins and re-folding or degradation of misfolded/aggregated proteins^3,5^. Functionally, Hsp70 display two domains, the substrate binding domain (SBD), which recognizes specific substrates or client proteins, and the nucleotide binding domain (NBD), which binds ATP and regulates client association through ATP hydrolysis. A short peptide links both domains and allows allosteric changes that modulate Hsp70 interaction with clients due to ATP/ADP cycles^3^. Whether Hsp70 is located extracellularly is not clear, but some reports claim both intracellular and extracellular roles for Hsp70^3,6,7^.

AD is the most prevalent neurodegenerative disorder; a dementia that particularly affects the aging population with a profound personal, medical and social impact. Hyperphosphorylated tau protein accumulation in intracellular neurofibrillary tangles (NTFs) and amyloid-β1-42 (Aβ42) peptide deposition in extracellular plaques are the two major hallmarks of AD^8^. As the original amyloid cascade hypothesis postulates, accumulation of Aβ42 constitute the triggering event in AD^9^, and recent updates to this hypothesis indicate that pre-amyloid structures are the most toxic Aβ42 species^10^. Sequential cleavage of the amyloid precursor protein (APP) by β-secretase (BACE1) and γ-secretase produces the Aβ42 peptide, which is then secreted to the extracellular space. Aβ42 can also be internalized by re-uptake^11^ and endocytosis^12^. Aβ42, either directly in the cytosol or from the extracellular space through binding to specific receptors (i.e.: NMDA, AMP, nAChR and mGluR5), promotes activation of several kinases, which include CaMKK2, JunK and GSK3^12^. These kinases eventually hyperphosphorylate tau, which has been proposed to be the executor of the pathogenic process, affecting synaptic function and leading to cognitive impairments.

In *Drosophila*, a number of AD models replicate relevant features of AD and, some exhibit memory impairments due to overexpression of tau^13–15^, hAPP, APP-like (APPL, the *Drosophila* orthologue of APP)^16,17^ or Aβ42^18^. Interestingly, ubiquitous expression of tau selectively affected the mushroom body (MB) neurons, which is consistent with the neuron-specific pathology of AD^14,15^. Furthermore, the mechanisms of memory formation seem to be more sensitive to expression of Aβ42 or Aβ40 than the mechanisms underlying locomotion, as memory deficits are observed in flies as young as 6 day-old, while locomotion remains unaltered until they reach the age of 20-day old^18^. In addition, expression of a human form of Aβ42 carrying the mutation associated with early-onset familial AD (E22G), EOFAD-related Arctic mutation (Aβ42Arc), induces an earlier onset of the memory deficiencies than that observed for wild type Aβ42^19^. This suggests that this memory assay, which employs olfactory classical conditioning in *Drosophila*, is particularly sensitive to clinically relevant variants of Aβ42.

In recent years, *Drosophila* models of cognition have been established as a standard system to explore the learning and memory mechanisms at the molecular, cellular, and behavioral levels. Studies in the past 3 decades has unraveled important parallelisms between *Drosophila* and mammals concerning the anatomy of the olfactory pathway, the organization of neuronal circuits and the signaling cascades underlying the formation of memories^20^. The *Drosophila* MBs are a bilateral structure located in the dorsal region of the brain and comprised of approximately two thousand neurons in each brain hemisphere, the Kenyon Cells. The axons of these neurons project ventrally to form the MB lobes, where they connect with a plethora of post-synaptic neurons through the release of acetylcholine. The neuronal activity that Kenyon cells orchestrate plays a major role in the formation and storage of olfactory memories^21–23^. The MB neurons in combination with the use of olfactory classical conditioning protocols are ideally suited to examine the effectiveness of novel therapeutic agents targeting the neurodegenerative actions of human amyloids, their efficiency, and functionality. As these amyloids are mostly secreted to the extracellular space, we engineered a molecular system that delivers a potential blocking agent, the Hsp70, to this extracellular region. We previously showed that secreted Hsp70 efficiently suppressed Aβ42 neurotoxicity through its holdase domain^24^. In that study, we monitored locomotor activity as the alternative physiological assay to evaluate neuronal activity. Although this method resulted effective in providing functional evidence, the paradigm was not relevant in the context of AD.

Here, we thoroughly characterized the age-dependent memory decline of flies expressing Aβ42 in the MB neurons and determined that expression of the amyloid in the *Drosophila* brain progressively abolishes the formation of new memories. Using this sensitive behavioral assay, we also observed that the onset of these memory deficits was dose-dependent; the higher the level of Aβ42, the earlier the onset of the memory impairments. We then demonstrated that both cytosolic and secreted forms of Hsp70 fully restored memory acquisition in flies expressing Aβ42, irrespective of their age. Although these two forms may function through independent pathways, they both alleviated the memory decline caused by Aβ42 accumulation without affecting that associated with aging. Altogether, the data confirm the relevance of this *Drosophila* model of AD in evaluating Aβ42-induced memory impairments and in searching for protective molecules and compounds with a prospective therapeutic application.

## Results

### Aβ42-induced memory impairments are proportional to amyloid expression level

We had previously developed a *Drosophila* model of AD^25^, in which we specifically expressed the *Aβ*42 construct^26^ in the mushroom body (MB) neurons and monitored memory acquisition throughout aging, and up to 30 days of age. Our fly model displayed severe memory impairments after olfactory classical conditioning^25^. As memory was drastically impaired since day 1 post-eclosion, we decided to lower the expression level of the *Aβ*42 construct^26^ and monitor the progression of these memory deficits under these new conditions. Gal4 thermostability enables controlling the cumulative amount of Gal4 by temperature, with a maximal level being reached at the optimal temperature of 30 °C^27^. Decreases in temperature from this optimal value reduce the level of Gal4 and, consequently, the transcriptional activity of the Gal4 over the UAS construct.

**Figure 1:**
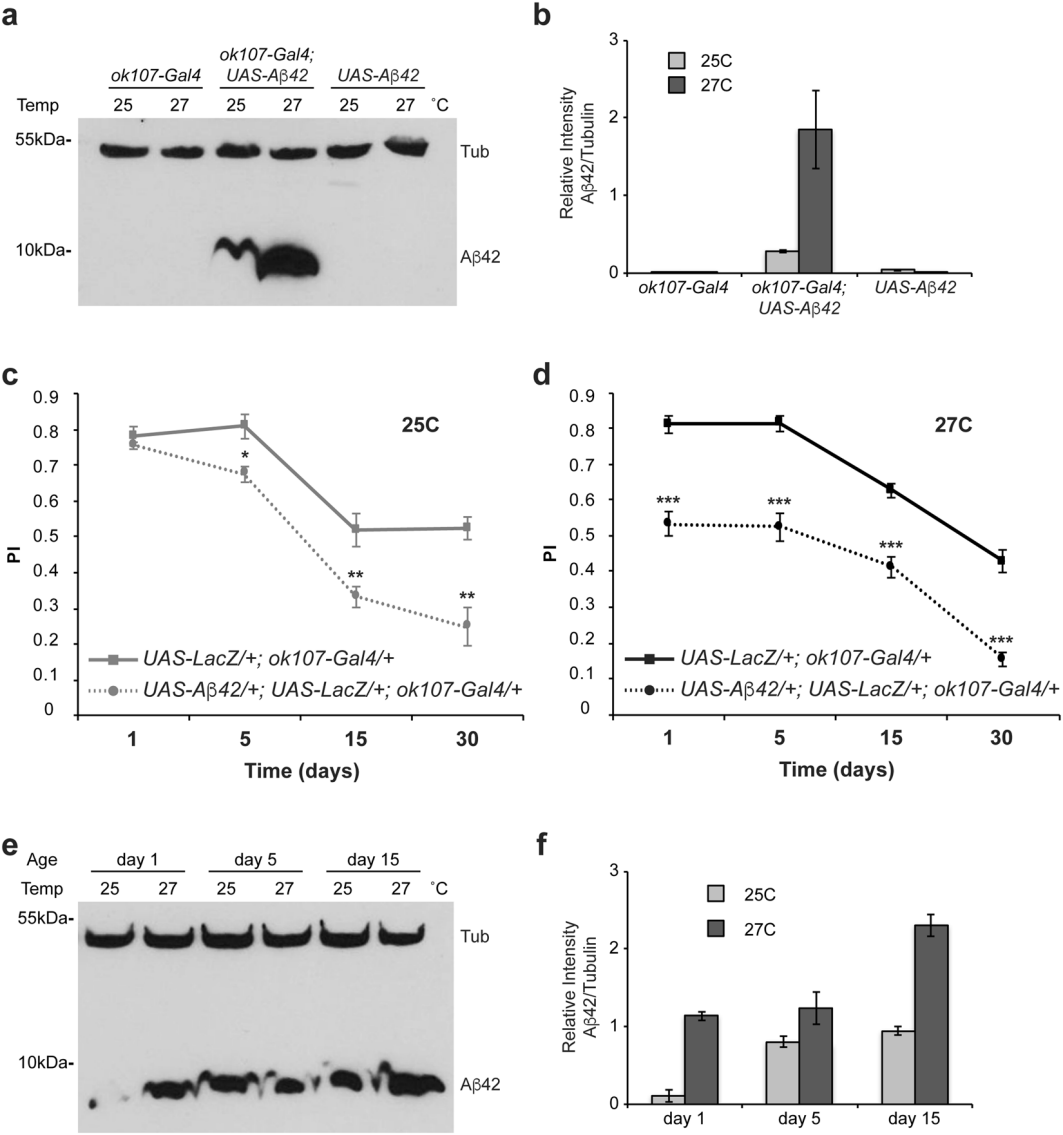
Aβ42 expression level dictates the onset of memory impairments in a *Drosophila* model of Alzheimer’s disease. (**a**) Protein extracts from heads of flies raised at 25 °C or 27 °C were immunodetected for Aβ42 and β-Tubulin. (**b**) Aβ42 levels were quantified and normalized to the amount of β-Tubulin for control flies (*UAS-LacZ*/+*; ok107-Gal4*/+ and *UAS-Aβ42*/+) and flies expressing Aβ42 in the MBs (*UAS-Aβ42*/+; *ok107-Gal4*/+) at both temperatures. (**c**,**d**) Flies were raised at either 25 °C (**c**) or 27 °C (**d**) throughout development and up to day 1, 5, 15 or 30 post-eclosion and then trained using olfactory classical conditioning. Flies were tested immediately after conditioning. Memory performance index (P.I.) is shown for control flies (*UAS-LacZ*/+; *ok107-Gal4*/+) and flies expressing Aβ42 in the MB neurons (*UAS-Aβ42*/+; *UAS-LacZ*/+*; ok107-Gal4*/+). (**c**) At 25 °C, control flies show normal memory decay through aging (day 1 vs day 5, p = 0.5202; day 5 vs day 15, p = 0.0005; day 15 vs day 30, p = 0.9215). Expression of the A*β*42 peptide in the MB neurons (*UAS-Aβ42*/+; *UAS-LacZ*/+*; ok107-Gal4*/+) enhances this memory decline (day 1 vs day 5, p = 0.1031; day 5 vs day 15, p = < 0.0001; day 15 vs day 30, p = 0.2099), but does not significantly impair memory performance until day 5 (t-test comparison; day 1, p = 0.6056; day 5, p = 0.0123; day 15, p = 0.0071; day 30, p = 0.0015) and then significantly increases with age. (**d**) At 27 °C, control flies show a memory decline associated with age that is slightly more pronounced at older ages, and this is significantly increased in flies expressing Aβ42 in the MB neurons (*UAS-Aβ42*/+; *UAS-LacZ*/+*; ok107-Gal4*/+*)* at all ages tested (t-test comparison; day 1, p < 0.0001; day 5, p < 0.0001; day 15, p = 0.0002; day 30, p < 0.0001). Error bars indicate SEM; n = 10 per group; ^*^p < 0.05, ^**^p < 0.001, ^***^p < 0.001. (**e**) Aβ42 immunodetection at day 1, 5 and 15 post-eclosion in protein extracts from flies (*UAS-Aβ42*/+; *ok107-Gal4*/+) raised at 25 °C or 27 °C. (**f**) Aβ42 levels were normalized to the amount of β-Tubulin in the matching groups, observing a correlation between temperature and the level of Aβ42 from day 1 to day 15 (n > 3).

Experiments carried out between 29 °C and 30 °C limited the amount of flies to perform these assays due to excessive lethality. Then, we tested whether a two-degree shift in temperature (27 °C to 25 °C) could lead to significant changes in the expression level of Aβ42. We detected a very small amount of Aβ42 in flies (*UAS-Aβ*42/+*; UAS-LacZ*/+; *ok107-Gal4*/+) raised at 25 °C when compared to those developed at 27 °C (Fig. 1a,b), while control lines carrying either the driver construct (*UAS-LacZ*/+*; ok107-Gal4*/+) or the effector element (*UAS-Aβ*42/+) alone did not trigger any expression of Aβ42. Hence, we evaluated memory acquisition in control flies (*UAS-LacZ*/+*; ok107-Gal4*/+) and flies expressing Aβ42 in the MBs (*UAS-Aβ*42/+*; UAS-LacZ*/+*; ok107-Gal4*/+) at 27 °C and 25 °C from day 1 to day 30 post-eclosion. In control flies, memory performance was statistically comparable at both temperatures (Supplementary Fig. 1). However, memory acquisition of flies expressing Aβ42 in the MBs became increasingly impaired as they aged at 25 C (Fig. 1c), but was consistently disrupted through all ages tested at 27 °C (Fig. 1d). At the higher temperature of 27 °C, memory acquisition was severely affected since day 1 post-eclosion and the level of impairment was maintained throughout all ages tested (Fig. 1d). This early phenotype was probably due to the high expression level of Aβ42 accrued during development (Fig. 1a,b). At 25 °C, conversely, no memory deficits were observed until day 5 post-eclosion (Fig. 1c); in fact, memory performance of flies expressing Aβ42 at 25 °C was not as impaired as in those at 27 °C until 15 days of age (Supplementary Fig. 1). By this time, Aβ42 levels at 25 °C were equivalent to those at 27 °C at day 1 (Fig. 1e,f). Nonetheless, Aβ42 expression was consistently higher at 27 °C throughout aging (Fig. 1e,f). By day 30, memory performance was drastically disrupted and at comparable levels irrespective of the temperature. Together, these results indicated that Aβ42 triggered a progressive cognitive decline whose onset is dependent on the amount of Aβ42.

**Figure 2:**
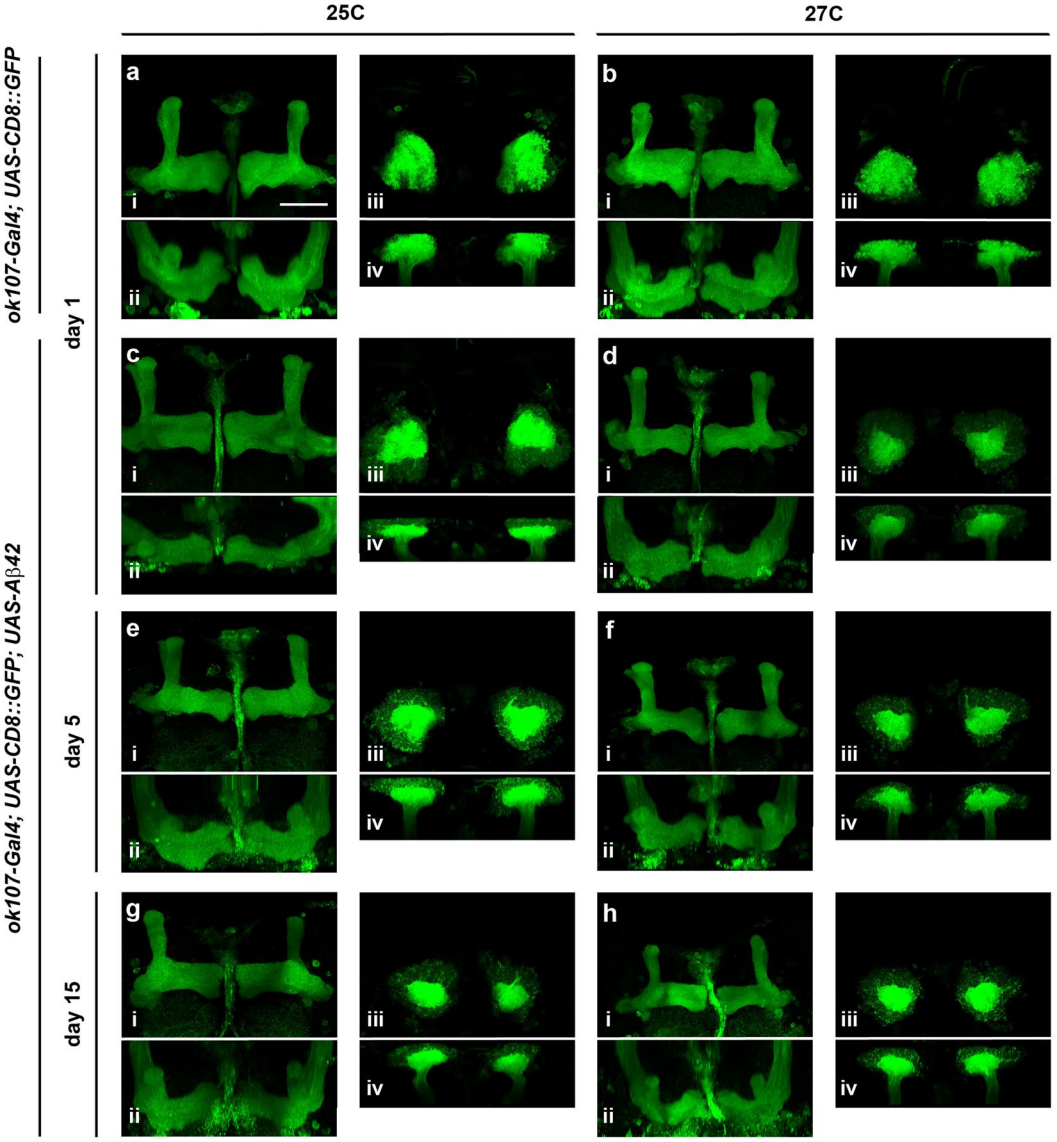
The architecture of the mushroom body is preserved in flies expressing Aβ42. Representative confocal images of the MBs from flies co-expressing CD8::GFP and LacZ (*UAS-LacZ*/+*; UAS-CD8::GFP*/+*; ok107-Gal4*/+) or Aβ42 (*UAS-Aβ42*/+; *UAS-CD8::GFP*/+*; ok107-Gal4*/+) in the MB neurons at day 1 (**a**–**d**), 5 (**e**,**f**) and 15 (**g**,**h**) post-eclosion. The morphology of the MBs was analyzed in three-dimensional reconstructions of brains from flies raised at 25 °C (**a**,**c**,**e**,**g**) and 27 °C (**b**,**d**,**f**,**h**). Frontal (**i**) and dorsal (**ii**) view of the MB lobes and dorsal (**iii**) and frontal (**iv**) view of the MB calyxes. No significant defects were found at the macroscopic level. Scale bar 30 μm.

Interestingly, despite the severity in the cognitive impairments observed, no significant morphological alterations were found in the MBs of flies expressing Aβ42 at the macroscopic level (Fig. 2). At 27 °C, however, the volume of the MB lobes (axonal) and, more prominently, the calyxes of the Kenyon Cells (somatic and dendritic) tended to shrink due to Aβ42-induced neurodegeneration; but this effect does not became significant until day 20 post-eclosion, as it was previously reported^25^. Remarkably, the memory impairments were present (i.e.: day 1 at 27 °C and day 5 at 25 °C) before any morphological defects became detectable (Fig. 2a,b,d,e).

### Expression of the Aβ42 peptide in the MBs does not disrupt stimuli perception

Given the drastic memory impairment observed in Aβ42 expressing flies (*UAS-Aβ*42/+*; UAS-LacZ*/+*; ok107-Gal4*/+), we tested whether the sensory stimuli presented to these flies was properly perceived, as this is a requirement for memories to be formed. Otherwise, memory deficits would be the result of a disrupted perception rather than a deficiency in the formation of memories *per se*. Thus, it was critical to exclude the possibility that these memory impairments were the result of abnormalities in stimuli perception. We then assessed shock and odor acuity of flies expressing Aβ42 in the MBs and compared them to those of control flies. Avoidance indexes for an electric shock of 90 V (Table 1 and Supplementary Table 1) and the two odors, octanol (Table 2 and Supplementary Table 2) and benzaldehyde (Table 3 and Supplementary Table 3), at the same working concentrations used for conditioning, did not reveal significant differences between controls and Aβ42 expressing flies. Nonetheless, we observed a small tendency for flies expressing Aβ42 to have a lower sensitivity to these odors at day 5 (Supplementary Tables 2–3). This was especially noticeable for benzaldehyde, in which case the tendency was present at day 30 as well (Supplementary Table 3). At day 15, however, shock and odor perception was identical to controls. Therefore, the memory impairments observed in flies expressing Aβ42 in the MB neurons (Fig. 1c,d) are caused by disruptions in the mechanism of memory formation rather than deficits in sensory perception.

**Table 1:** Shock avoidance of flies co-expressing the Hsp70 isoforms and Aβ42.

Genotype	90 V Shock Avoidance	*p-value*
*UAS-LacZ*/+*; ok107-Gal4*/+	0.6744 ± 0.0513	—
*UAS-Aβ42*/+*; UAS-LacZ*/+*; ok107-Gal4*/+	0.6241 ± 0.0599	0.9926
*UAS-Aβ42*/*UAS-secHsp70; ok107-Gal4*/+	0.7034 ± 0.0601	0.9998
*UAS-Aβ42*/*UAS-cytHsp70; ok107-Gal4*/+	0.8012 ± 0.0426	0.6946
*UAS-Aβ42*/+*; UAS-LacZ*/+*;* +/+	0.6592 ± 0.0695	>0.9999
*UAS-Aβ42*/*UAS-secHsp70;* +/+	0.7891 ± 0.0316	0.7823
*UAS-Aβ42*/*UAS-cytHsp70;* +/+	0.7928 ± 0.0336	0.7572
*UAS-secHsp70*/+*; ok107-Gal4*/+	0.7034 ± 0.0429	0.9763
*UAS-cytHsp70*/+*; ok107-Gal4*/+	0.6893 ± 0.0294	0.9458

Avoidance to an electric shock of 90 Volts for each corresponding genotype and their Tukey’s comparison test *p-value* for statistical significance versus the control (*UAS-LacZ*/+*; ok107*-*Gal4*/+).

**Table 2:** Odor avoidance for 3-octanol of flies co-expressing Hsp70 isoforms and Aβ42.

Genotype	Octanol Avoidance	*p-value*
*UAS-LacZ*/+*; ok107-Gal4*/+	0.8116 ± 0.0121	—
*UAS-Aβ42*/+*; UAS-LacZ*/+*; ok107-Gal4*/+	0.5096 ± 0.1088	0.0530
*UAS-Aβ42*/*UAS-secHsp70; ok107-Gal4*/+	0.8422 ± 0.0360	0.9996
*UAS-Aβ42*/*UAS-cytHsp70; ok107-Gal4*/+	0.7110 ± 0.0493	0.8323
*UAS-Aβ42*/+*; UAS-LacZ*/+*;* +/+	0.7227 ± 0.0539	0.8979
*UAS-Aβ42*/*UAS-secHsp70;* +/+	0.8663 ± 0.0352	0.9902
*UAS-Aβ42*/*UAS-cytHsp70;* +/+	0.8873 ± 0.0107	0.9502
*UAS-secHsp70*/+*; ok107-Gal4*/+	0.7852 ± 0.0396	0.9483
*UAS-cytHsp70*/+*; ok107-Gal4*/+	0.7948 ± 0.0403	0.9932

Avoidance to the odor 3-octanol for each corresponding genotype and their Tukey’s comparison test *p-value* for statistical significance versus the control (*UAS-LacZ*/+; *ok107-Gal4*/+).

**Table 3:** Odor avoidance for benzaldehyde of flies co-expressing the Hsp70 isoforms and Aβ42.

Genotype	Benzaldehyde Avoidance	*p-value*
*UAS-LacZ*/+*; ok107-Gal4*/+	0.3944 ± 0.0805	—
*UAS-Aβ42*/+*; UAS-LacZ*/+*; ok107-Gal4*/+	0.1420 ± 0.1144	0.2999
*UAS-Aβ42*/*UAS-secHsp70; ok107-Gal4*/+	0.1597 ± 0.0489	0.3764
*UAS-Aβ42*/*UAS-cytHsp70; ok107-Gal4*/+	0.2035 ± 0.0803	0.3208
*UAS-Aβ42*/+*; UAS-LacZ*/+*;* +/+	0.0680 ± 0.1193	0.0937
*UAS-Aβ42*/*UAS-secHsp70;* +/+	0.2269 ± 0.0552	0.7187
*UAS-Aβ42*/*UAS-cytHsp70;*+/+	0.2765 ± 0.0537	0.9160
*UAS-secHsp70*/+*; ok107-Gal4*/+	0.2837 ± 0.0702	0.9483
*UAS-cytHsp70*/+*; ok107-Gal4*/+	0.3062 ± 0.0926	0.9262

Avoidance to the odor benzaldehyde for each corresponding genotype and their Turkey’s comparison test p*-value* for statistical significance versus the control (*UAS-LacZ*/+; *ok107-Gl4*/+).

### Secreted Hsp70 protects against Aβ42-mediated memory deficits in *Drosophila*

**Figure 3:**
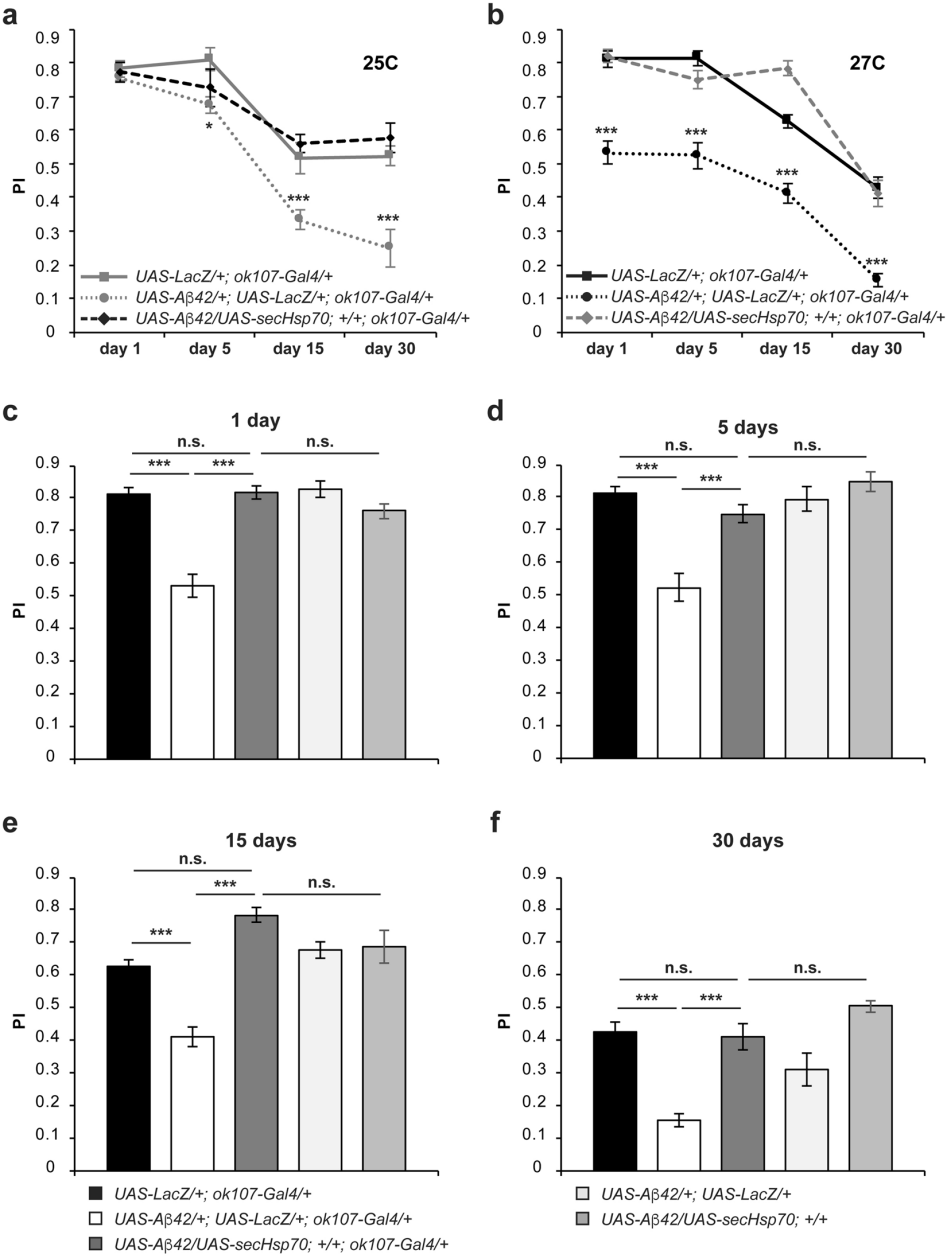
Expression of the secreted Hsp70 prevents memory impairments in a *Drosophila* model of Alzheimer’s disease. Flies were raised at either 25 °C (**a**) or 27 °C (**b**–**f**) throughout development and up to day 1 (**c**), 5 (**d**), 15 (**e**) or 30 (**f**) post-eclosion and then trained using olfactory classical conditioning. Flies were tested immediately after conditioning. Memory performance index (P.I.) is shown for control flies (*UAS-LacZ*/+*; ok107-Gal4*/+*)*, flies expressing Aβ42 and LacZ (*UAS-Aβ42*/+; *UAS-LacZ*/+*; ok107-Gal4*/+*)* or in combination with the secreted form of Hsp70 (*UAS-Aβ42*/+; *UAS-secHsp70*/+*; ok107-Gal4*/+*)* and control flies without carrying the Gal4 driver (*UAS-Aβ42*/+; *UAS-LacZ*/+ and *UAS-Aβ42*/+; *UAS-secHsp70*/+*)*. (**a**) At 25 °C, expression of the secreted form of Hsp70 in the MB neurons fully rescues the memory impairments caused by accumulation of Aβ42 in this set of neurons (*UAS-Aβ42*/+; *UAS-LacZ*/+*; ok107-Gal4*/+vs *UAS-Aβ42*/+; *UAS-secHsp70*/+*; ok107-Gal4*/+; day 1, p = 0.9833; day 5, p = 0.8109; day 15, p = 0.0043; day 30, p = 0.0001); to scores essentially equivalent to those of control flies (*UAS-LacZ*/+; *ok107-Gal4*/+ vs *UAS-Aβ42*/+; *UAS-secHsp70*/+*; ok107-Gal4*/+; day 1, p = 0.9901; day 5, p = 0.4367; day 15, p = 0.8839; day 30, p = 0.8084). (**b**) At 27 °C, expression of the secreted Hsp70 chaperone in the MB neurons rescues the memory deficits caused by Aβ42 deposition (*UAS-Aβ42*/+; *UAS-LacZ*/+*; ok107-Gal4*/+ vs *UAS-Aβ42*/+; *UAS-secHsp70*/+*; ok107-Gal4*/+; day 1, p < 0.0001; day 5, p < 0.0001; day 15, p < 0.0001; day 30, p = 0.0002). Memory performance of flies expressing secHsp70 (*UAS-Aβ42*/+; *UAS-secHsp70*/+*; ok107-Gal4*/+) is equivalent to that of control flies (*UAS-LacZ*/+*; ok107-Gal4*/+) at all ages tested (day 1, p > 0.9999; day 5, p = 0.7065; day 15, p = 0.0529; day 30, p > 0.9999). (**c**) One-day-old flies expressing Aβ42 and LacZ in the MB neurons display a significantly lower memory performance (*UAS-Aβ42*/+; *UAS-LacZ*/+*; ok107-Gal4*/+, p < 0.0001) than control flies expressing LacZ alone (*UAS-LacZ*/+*; ok107-Gal4*/+). Flies co-expressing the secreted form of Hsp70 and Aβ42 (*UAS-Aβ42*/+; *UAS-secHsp70*/+*; ok107-Gal4*/+) performed at a significantly higher level than flies co-expressing Aβ42 and LacZ (*UAS-Aβ42*/+; *UAS-LacZ*/+*; ok107-Gal4*/+, p < 0.0001) and their memory performance was statistically undistinguishable from control flies (*UAS-LacZ*/+*; ok107-Gal4*/+, p > 0.9999; *UAS-Aβ42*/+; *UAS-LacZ*/+, p > 0.9999; *UAS-Aβ42*/+; *UAS-secHsp70*/+, p = 0.7506). (**d**) Five-day-old flies expressing Aβ42 and LacZ in the MB neurons display a significantly lower memory performance (p < 0.0001) than control flies expressing LacZ alone. Flies co-expressing the secreted Hsp70 and Aβ42 performed at a significantly higher level than flies co-expressing Aβ42 and LacZ (p < 0.0001) and their memory performance was statistically undistinguishable from control flies (*UAS-LacZ*/+*; ok107-Gal4*/+, p = 0.7065; *UAS-Aβ42*/+; *UAS-LacZ*/+, p = 9283; *UAS-Aβ42*/+; *UAS-secHsp70*/+, p = 2109). (**e**) Fifteen-day-old flies expressing Aβ42 and LacZ in the MB neurons display a significantly lower memory performance (p < 0.0003) than control flies expressing LacZ alone. Flies co-expressing the secreted Hsp70 and Aβ42 performed at a significantly higher level than flies co-expressing Aβ42 and LacZ (p < 0.0001) and their memory performance was statistically undistinguishable from control flies (*UAS-LacZ*/+*; ok107-Gal4*/+, p = 0.0529; *UAS-Aβ42*/+; *UAS-LacZ*/+, p = 0.2066; *UAS-Aβ42*/+; *UAS-secHsp70*/+, p = 0.3045). (**f**) Thirty-days-old flies expressing Aβ42 and LacZ in the MB neurons display a significantly lower memory performance (p < 0.0001) than control flies expressing LacZ alone. Flies co-expressing the secreted form of Hsp70 and Aβ42 performed at a significantly higher level than flies co-expressing Aβ42 and LacZ (p = 0.0002) and their memory performance was statistically undistinguishable from control flies (*UAS-LacZ*/+*; ok107-Gal4*/+, p > 0.9999; *UAS-Aβ42*/+; *UAS-LacZ*/+, p = 0.4083; *UAS-Aβ42*/+; *UAS-secHsp70*/+, p = 0.5252). Error bars indicate SEM; n = 10 per group; Tukey’s comparison test: *p < 0.05, ***p < 0.001.

Once we analyzed the dynamics of the Aβ42-mediated memory decline in *Drosophila*, we tested the ability of the heat shock protein 70 (Hsp70) to protect and/or prevent flies from the memory impairments caused by Aβ42 neurotoxicity. The Hsp70 is a molecular chaperone with demonstrated neuroprotective activity against intracellular amyloids that can also tackle extracellular targets through genetic engineering^24^. As Aβ42 is secreted to the extracellular space, we first employed the secreted form of the Hsp70 (secHsp70) at both previously tested temperatures, 25 °C and 27 °C. Flies developed at 25 °C and co-expressing the secHsp70 and Aβ42 in the MB neurons (*UAS-Aβ*42/*UAS-secHsp70*; *ok107-Gal4*/+) performed significantly higher than flies co-expressing Aβ42 and LacZ (*UAS-Aβ*42/+; *UAS-LacZ*/+*; ok107-Gal4*/+) at day 15 and 30 post-eclosion, and statistically equivalent to the corresponding control group (*UAS-LacZ*/+*; ok107-Gal4*/+) at day 1, 5, 15 and 30 post-eclosion (Fig. 3a). Although performance of flies co-expressing Aβ42 and secHsp70 was always equivalent to that of control flies, memory performance of flies co-expressing Aβ42 and LacZ was also very similar to that of flies co-expressing Aβ42 and secHsp70 at day 1 and 5 (Fig. 3a). Given that the lower level of Aβ42 at this temperature led to a milder memory deficit (Supplementary Fig. 1), we next asked whether secHsp70 would protect against cognitive deficits triggered by a higher load of Aβ42.

We then performed the same experimental approach at 27 °C. At this temperature, flies co-expressing the secHsp70 and Aβ42 in the MB neurons (*UAS-Aβ*42/*UAS-secHsp70*; *ok107-Gal4*/+) performed significantly higher than flies co-expressing Aβ42 and LacZ (*UAS-Aβ*42/+; *UAS-LacZ*/+*; ok107-Gal4*/+), and comparable to the respective control group (*UAS-LacZ*/+*; ok107-Gal4*/+) at day 1, 5, 15 and 30 post-eclosion (Fig. 3b). Further, memory acquisition of one- (Fig. 3c), five- (Fig. 3d), fifteen- (Fig. 3e) and thirty-day-old (Fig. 3f) flies co-expressing secHsp70 and Aβ42 was statistically equivalent to that of the corresponding control groups: flies expressing LacZ alone (*UAS-LacZ*/+*; ok107-Gal4*/+), flies carrying the *secHsp70* and *Aβ*42 (*UAS-Aβ*42/*UAS-secHsp70*) transgenes or the *Aβ42* (*UAS-Aβ42*/*UAS-LacZ*) in the absence of Gal4 driver, and flies expressing the secHsp70 alone (*UAS-secHsp70*/+*; ok*1*07-Gal4*/+) (Tables 4–7).

**Table 4:** Tukey’s comparison test *p-values* at day 1.

Genotypes	1	2	3	4	5	6	7	8	9
1	*—*	<*0*.*0001*	>*0*.*9999*	>*0*.*9999*	*0*.*9994*	*0*.*8586*	*0*.*9*3*05*	*0*.*0550*	*0*.*1339*
2	*—*	*—*	*<0*.*0001*	*<0*.*0001*	*<0*.*0001*	*<0*.*0001*	*<0*.*0001*	*<0*.*0001*	*<0*.*0001*
3	*—*	*—*	*—*	>*0*.*9999*	>*0*.*9999*	*0*.*7506*	*0*.*9753*	*0*.*8028*	*0*.*7983*
4	*—*	*—*	*—*	*—*	>*0*.*9999*	*0*.*7*5*37*	*0*.*9871*	*0*.*9289*	*0*.*793*6
5	*—*	*—*	*—*	*—*	*—*	*0*.*6156*	*0*.*994*7	*0*.*9346*	*0*.8*5*9*2*
6	*—*	*—*	*—*	*—*	*—*	*—*	*0*.*2500*	*0*.*8592*	*0*.*9657*
7	*—*	*—*	*—*	*—*	*—*	*—*	*—*	*0*.*8567*	*0*.*8976*
8	*—*	*—*	*—*	*—*	*—*	*—*	*—*	*—*	*0*.*5340*
9	*—*	*—*	*—*	*—*	*—*	*—*	*—*	*—*	*—*

Table shows *p*-values for memory performance between paired genotypes at day 1. Genotypes are 1- *UAS-LacZ*/+; *ok107*-*Gal4*/+, 2- UAS-Aβ42/+; *UAS-LacZ*/+; *ok107-Gal4*/+, *3*- *UAS-Aβ42*/*UAS-secHsp70*; *ok107-Gal4*/+, 4- *UAS-Aβ42*/*UAS-cytHsp70*; *ok107-Gal4*/+, 5- *UAS-Aβ42*/+; *UAS-LacZ*/+*;*+/+, 6- *UAS-Aβ42*/*UAS-secHsp70*;+/+, 7- *UAS-Aβ42*/*UAS-cytHsp70*;+/+, 8- *UAS-secHsp70*/+; *ok107-Gal4*/+, and 9- *UAS-cytHsp70*/+; *ok107-Gal4*/+.

**Table 5:** Tukey’s comparison test p-values at day 5.

Genotypes	1	2	3	4	5	6	7	8	9
1	*—*	*<0*.*0001*	*0*.*7065*	*0*.*9963*	*0*.*9990*	*0*.*9728*	*0*.*9686*	*0*.*2960*	*0*.*7947*
2	*—*	*—*	*<0*.*0001*	*<0*.*0001*	*<0*.*0001*	*<0*.*0001*	*<0*.*0001*	*<0*.*0001*	*<0*.*0001*
3	*—*	*—*	*—*	*0*.*9593*	*0*.*9283*	*0*.*2109*	*0*.*2010*	*0*.*8394*	*0*.*8937*
4	*—*	*—*	*—*	*—*	>*0*.*9999*	*0*.*7513*	*0*.*7262*	*0*.*9283*	*0*.*9721*
5	*—*	*—*	*—*	*—*	*—*	*0*.*8188*	*0*.*8055*	*0*.*9562*	*0*.*9523*
6	*—*	*—*	*—*	*—*	*—*	*—*	>*0*.*9999*	*0*.*9835*	*0*.*9362*
7	*—*	*—*	*—*	*—*	*—*	*—*	*—*	*0*.*8873*	*0*.*9284*
8	*—*	*—*	*—*	*—*	*—*	*—*	*—*	*—*	*0*.*8943*
9	*—*	*—*	*—*	*—*	*—*	*—*	*—*	*—*	*—*

Table shows *p*-values for memory performance between paired genotypes at day 5. Genotypes are 1- *UAS-LacZ*/+; *ok107-Gal4*/+, *2- UAS-Aβ42*/+; *UAS-LacZ*/+; *ok107-Gal4*/+, *3- UAS-Aβ42*/*UAS-secHsp70*; *ok107-Gal4*/+, *4- UAS-Aβ42*/*UAS-cytHsp70*; *ok107-Gal4*/+, *5- UAS-Aβ42*/+; *UAS-LacZ*/+*;*+/+, *6- UAS-Aβ42*/*UAS-secHsp70;*+/+, *7- UAS-Aβ42*/*UAS-cytHsp70;*+/+, *8- UAS-secHsp70*/+; *ok107-Gal4*/+, *and 9- UAS-cytHsp70*/+; *ok107-Gal4*/+.

**Table 6:** Tukey’s comparison test *p-values* at day 15.

Genotypes	1	2	3	4	5	6	7	8	9
1	*—*	*0*.*0003*	*0*.*0529*	*0*.*4423*	*0*.*8845*	*0*.*7791*	*0*.*6855*	*0*.*4265*	*0*.*6182*
2	*—*	*—*	*<0*.*0001*	*<0*.*0001*	*<0*.*0001*	*<0*.*0001*	*<0*.*0001*	*<0*.*0001*	*<0*.*0001*
3	*—*	*—*	*—*	*0*.*6301*	*0*.*2066*	*0*.*3045*	*0*.*3903*	*0*.*7384*	*0*.*6928*
4	*—*	*—*	*—*	*—*	*0*.*9863*	*0*.*9978*	*0*.*9997*	*0*.*9924*	*0*.*9268*
5	*—*	*—*	*—*	*—*	*—*	>*0*.*9999*	*0*.*9997*	*0*.*9993*	*0*.*9385*
6	*—*	*—*	*—*	*—*	*—*	*—*	>*0*.*9999*	*0*.*9998*	*0*.*9953*
7	*—*	*—*	*—*	*—*	*—*	*—*	*—*	*0*.*9926*	*0*.*9981*
8	*—*	*—*	*—*	*—*	*—*	*—*	*—*	*—*	*0*.*9459*
9	*—*	*—*	*—*	*—*	*—*	*—*	*—*	*—*	*—*

Table shows p-values for memory performance between paired genotypes at day 15. Genotypes are 1- *UAS-LacZ*/+; *ok107-Gal4*/+, *2- UAS-Aβ42*/+; *UAS-LacZ*/+; *ok107-Gal4*/+, *3- UAS-Aβ42*/*UAS-secHsp70*; *ok107-Gal4*/+, *4- UAS-Aβ42*/*UAS-cytHsp70*; *ok107-Gal4*/+, *5- UAS-Aβ42*/+; *UAS-LacZ*/+; +/+, *6- UAS-Aβ42*/*UAS-secHsp70*; +/+, *7- UAS-Aβ42*/*UAS-cytHsp70*; +/+, *8- UAS-secHsp70*/+; *ok107-Gal4*/+, *and 9- UAS-cytHsp70*/+; *ok107-Gal4*/+.

**Table 7:** Tukey’s comparison test p-values at day 30.

Genotypes	1	2	3	4	5	6	7	8	9
1	*—*	*<0*.*0001*	>*0*.*9999*	>*0*.*9999*	*0*.*2542*	*0*.*7130*	>*0*.*9999*	*0*.*5554*	*0*.*8622*
2	*—*	*—*	*0*.*0002*	*0*.*0002*	*0*.*0413*	*<0*.*0001*	*<0*.*0001*	*<0*.*0001*	*<0*.*0001*
3	*—*	*—*	*—*	>*0*.*9999*	*0*.*4083*	*0*.*5252*	>*0*.*9999*	*0*.*8937*	*0*.*9936*
4	*—*	*—*	*—*	*—*	*0*.*3980*	*0*.*5364*	>*0*.*9999*	*0*.*7381*	*0*.*9825*
5	*—*	*—*	*—*	*—*	*—*	*0*.*0571*	*0*.*2546*	*0*.*4286*	*0*.*6038*
6	*—*	*—*	*—*	*—*	*—*	*—*	*0*.*7126*	*0*.*7538*	*0*.*7946*
7	*—*	*—*	*—*	*—*	*—*	*—*	*—*	*0*.*8026*	*0*.*8482*
8	*—*	*—*	*—*	*—*	*—*	*—*	*—*	*—*	*0*.*9299*
9	*—*	*—*	*—*	*—*	*—*	*—*	*—*	*—*	*—*

Table shows p-values for memory performance between paired genotypes at day 30. Genotypes are 1- *UAS-LacZ*/+; *ok107-Gal4*/+, *2- UAS-Aβ42*/+; *UAS-LacZ*/+; *ok107-Gal4*/+, *3- UAS-Aβ42*/*UAS-secHsp70*; *ok107-Gal4*/+, *4- UAS-Aβ42*/*UAS-cytHsp70*; *ok107-Gal4*/+, *5- UAS-Aβ42*/+; *UAS-LacZ*/+*;*+/+, *6- UAS-Aβ42*/*UAS-secHsp70;*+/+, *7- UAS-Aβ42*/*UAS-cytHsp70;*+/+, *8- UAS-secHsp70*/+; *ok107-Gal4*/+, *and 9- UAS-cytHsp70*/+; *ok107-Gal4*/+.

Additionally, electric shock avoidance (Table 1) and odor avoidance to octanol (Table 2) and benzaldehyde (Table 3) of flies co-expressing secHsp70 and Aβ42 and control flies carrying the *secHsp70* and *Aβ42* transgenes without Gal4 driver were both at the same level than those of the control group (*UAS-LacZ*/+*; ok*1*07-Gal4*/+), proving that secHsp70 and its combination with Aβ42 did not have effects in perception of the stimuli presented. Together, these results provided evidence of the protective effect of the secreted form of Hsp70 against the memory impairments caused by extracellular Aβ42 neurotoxicity. Regardless of the level of deleterious Aβ42 or the age of the individuals tested, secHsp70 was found to be an extraordinarily potent agent against Aβ42-mediated memory decline.

### Cytosolic Hsp70 protects against Aβ42-mediated memory deficits in *Drosophila*

**Figure 4:**
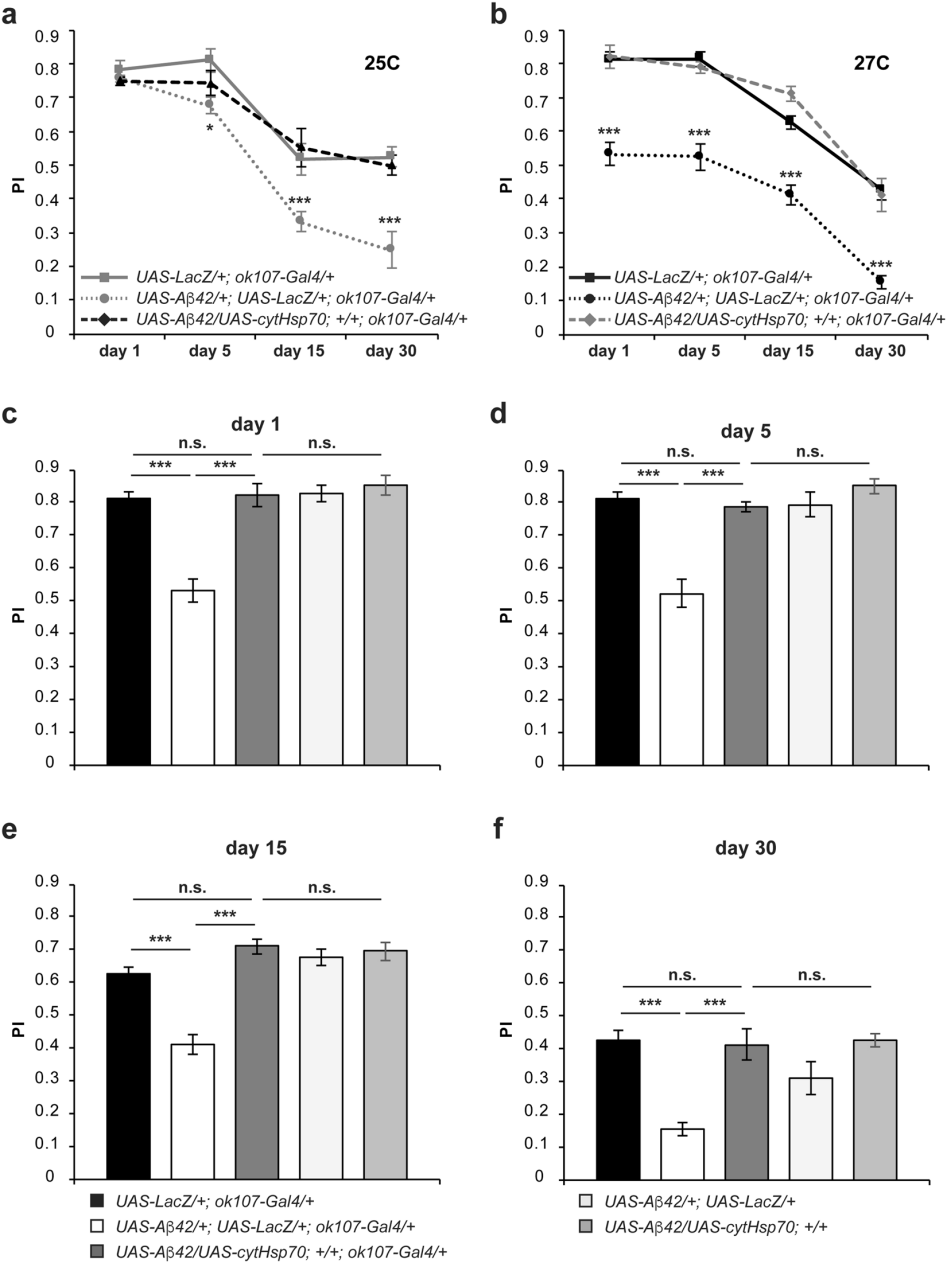
Expression of the cytosolic Hsp70 prevents memory impairments in a *Drosophila* model of Alzheimer’s disease. Flies were raised at either 25 °C (**a**) or 27 °C (**b–f**) throughout development and up to day 1 (**c**), 5 (**d**), 15 (**e**) or 30 (**f**) post-eclosion and then trained using olfactory classical conditioning. Flies were tested immediately after conditioning. Memory performance index (P.I.) is shown for control flies (*UAS-LacZ*/+*; ok107-Gal4*/+*)*, flies expressing Aβ42 and LacZ (*UAS-Aβ42*/+; *UAS-LacZ*/+*; ok107-Gal4*/+*)* or in combination with the cytosolic form of Hsp70 (*UAS-Aβ42*/+; *UAS-cytHsp70*/+*; ok107-Gal4*/+*)* and control flies without carrying the Gal4 driver (*UAS-Aβ42*/+; *UAS-LacZ*/+ and *UAS-Aβ42*/+; *UAS-cytHsp70*/+*)*. (**a**) At 25 °C, expression of the cytosolic Hsp70 in the MB neurons rescues the memory impairments caused by accumulation of Aβ42 in these neurons (*UAS-Aβ42*/+; *UAS-LacZ*/+*; ok107-Gal4*/+ vs *UAS-Aβ42*/+; *UAS-cytHsp70*/+*; ok107-Gal4*/+; day 1, p = 0.9991; day 5, p = 0.6834; day 15, p = 0.0083; day 30, p = 0.0039); to scores essentially equivalent to those of control flies (*UAS-LacZ*/ + ; *ok107-Gal4*/ + vs *UAS-Aβ42*/+; *UAS-secHsp70*/+*; ok107-Gal4*/+; day 1, p = 0.7899; day 5, p = 0.6424; day 15, p = 0.9373; day 30, p = 0.9784). (**b**) At 27 °C, expression of the cytosolic Hsp70 in the MB neurons rescues the memory deficits caused by Aβ42 deposition in this set of neurons (*UAS-Aβ42*/+; *UAS-LacZ*/+*; ok107-Gal4*/+ vs *UAS-Aβ42*/+; *UAS-cytHsp70*/+*; ok107-Gal4*/+; day 1, p < 0.0001; day 5, p < 0.0001; day 15, p < 0.0001; day 30, p = 0.0002). Memory performance of flies expressing cytHsp70 (*UAS-Aβ42*/+; *UAS-cytHsp70*/+*; ok107-Gal4*/+) is equivalent to that of control flies (*UAS-LacZ*/+*; ok107-Gal4*/+) at all ages tested (day 1, p > 0.9999; day 5, p = 0.9963; day 15, p = 0.4423; day 30, p > 0.9999). (**c**) One-day-old flies expressing Aβ42 and LacZ in the MB neurons display a significantly lower memory performance (*UAS-Aβ42*/+; *UAS-LacZ*/+*; ok107-Gal4*/+, p < 0.0001) than control flies expressing LacZ alone (*UAS-LacZ*/+*; ok107-Gal4*/+). Flies co-expressing the cytosolic Hsp70 and Aβ42 (*UAS-Aβ42*/+; *UAS-cytHsp70*/+*; ok107-Gal4*/+) performed at a significantly higher level than flies co-expressing Aβ42 and LacZ (*UAS-Aβ42*/+; *UAS-LacZ*/+*; ok107-Gal4*/+, p < 0.0001) and their memory performance was statistically undistinguishable from control flies (*UAS-LacZ*/+*; ok107-Gal4*/+, p > 0.9999; *UAS-Aβ42*/+; *UAS-LacZ*/+, p > 0.9999; *UAS-Aβ42*/+; *UAS-cytHsp70*/+, p = 0.9871). (**d**) Five-day-old flies expressing Aβ42 and LacZ in the MB neurons display a significantly lower memory performance (p < 0.0001) than control flies expressing LacZ alone. Flies co-expressing cytosolic Hsp70 and Aβ42 performed at a significantly higher level than flies co-expressing Aβ42 and LacZ (p < 0.0001) and their memory performance was statistically undistinguishable from control flies (*UAS-LacZ*/+*; ok107-Gal4*/+, p = 0.9963; *UAS-Aβ42*/+; *UAS-LacZ*/+, p > 0.9999; *UAS-Aβ42*/+; *UAS-cytHsp70*/+, p = 0.7362). (**e**) Fifteen-day-old flies expressing Aβ42 and LacZ in the MB neurons display a significantly lower memory performance (p < 0.0001) than control flies expressing LacZ alone. Flies co-expressing the cytosolic form of Hsp70 and Aβ42 performed at a significantly higher level than flies co-expressing Aβ42 and LacZ (p < 0.0001) and their memory performance was statistically undistinguishable from control flies (*UAS-LacZ*/+*; ok107-Gal4*/+, p = 0.4423; *UAS-Aβ42*/+; *UAS-LacZ*/+, p = 0.9863; *UAS-Aβ42*/+; *UAS-cytHsp70*/+, p = 0.9997). (**f**) Thirty-days-old flies expressing Aβ42 and LacZ in the MB neurons display a significantly lower memory performance (p < 0.0001) than control flies expressing LacZ alone. Flies co-expressing the cytosolic Hsp70 and Aβ42 performed at a significantly higher level than flies co-expressing Aβ42 and LacZ (p = 0.0002) and their memory performance was statistically undistinguishable from control flies (*UAS-LacZ*/+*; ok107-Gal4*/+, p > 0.9999; *UAS-Aβ42*/+; *UAS-LacZ*/+, p = 0.3980; *UAS-Aβ42*/+; *UAS-cytHsp70*/+, p > 0.9999). Error bars indicate SEM; n = 10 per group; Tukey’s comparison test: *p < 0.05, ***p < 0.001.

The secreted form of Hsp70 contains a signal peptide that ensures delivery to the extracellular space, where it can meet Aβ42 and mask the associated neurotoxicity. However, we have previously found that the cytosolic Hsp70 also protected against Aβ42-induced neuronal death in flies^24^, and others found additional protective effects in mice^6^. In order to test whether the cytosolic Hsp70 prevents the memory impairments triggered by Aβ42 neurotoxicity, we next employed the cytosolic form of Hsp70 (cytHsp70) at both previously tested temperatures, 25 °C and 27 °C. At 25 C, flies co-expressing the cytHsp70 and Aβ42 in the MB neurons (*UAS-Aβ42*/*UAS-cytHsp70*; *ok*1*07-Gal4*/+) performed significantly higher than flies co-expressing Aβ42 and LacZ (*UAS-Aβ42*/+; *UAS-LacZ*/+*; ok*1*07-Gal4*/+) at day 15 and 30 post-eclosion, and statistically equivalent to the corresponding control group (*UAS-LacZ*/+*; ok*1*07-Gal4*/+) at day 1, 5, 15 and 30 post-eclosion (Fig. 4a). At this lower temperature of 25 °C, the memory deficit of flies co-expressing Aβ42 and LacZ were not significantly different from those of flies co-expressing Aβ42 and secHsp70 at day 1 and 5 (Fig. 4a). As the lower levels of Aβ42 at this temperature lead to a milder cognitive decline, with no memory deficit at day 1 and 5 (Supplementary Fig. 1), we then carried out the same experimental procedure at 27 °C to test whether cytHsp70 protects against memory impairments triggered by a higher load of Aβ42.

At the temperature of 27 °C, flies co-expressing the cytHsp70 and Aβ42 in the MB neurons (*UAS-Aβ*42/*UAS-cytHsp70*; *ok*1*07-Gal4*/+) also performed significantly higher than flies co-expressing Aβ42 and LacZ (*UAS-Aβ4*2/+; *UAS-LacZ*/+*; ok107-Gal4*/+), and comparable to the respective control group (*UAS-LacZ*/+*; ok107-Gal4*/+) at day 1, 5, 15 and 30 post-eclosion (Fig. 4b). Further, memory acquisition of one- (Fig. 4c), five- (Fig. 4d), fifteen- (Fig. 4e) and thirty-day-old (Fig. 4f) flies co-expressing cytHsp70 and Aβ42 was statistically equivalent to that of the respective control groups: flies expressing LacZ alone (*UAS-LacZ*/+*; ok107-Gal4*/+), flies carrying the *cytHsp70* and *Aβ42* transgenes in the absence of Gal4 driver (*UAS-Aβ42*/*UAS-cytHsp70*) or the *Aβ42* transgene alone (*UAS-Aβ42*/*UAS-LacZ*), and flies expressing the cytHsp70 alone (*UAS-cytHsp70*/+*; ok107-Gal4*/+) (Tables 4–7).

Additionally, electric shock avoidance (Table 1) and odor avoidance to octanol (Table 2) and benzaldehyde (Table 3) of flies co-expressing cytHsp70 and Aβ42 and the control group carrying the *cytHsp70* and *Aβ42* transgenes without Gal4 driver were both at the same level than those of the control group (*UAS-LacZ*/+*; ok107-Gal4*/+), demonstrating that cytHsp70 or its combination with Aβ42 had no effects in perception of the stimuli presented. These findings were consistent with previous studies in mice^6^ and provided evidence of the intracellular function that Hsp70 exerts protecting against the memory impairments caused by Aβ42 neurotoxicity. Together, the data presented here revealed an effective prevention of the Aβ42-triggered cognitive deficiencies via Hsp70 activity, from the cytosol as well as from the extracellular space, irrespective of the Aβ42 load or the age of the individuals affected.

### Hsp70 does not enhance memory acquisition either in the cytosol or extracellularly

**Figure 5:**
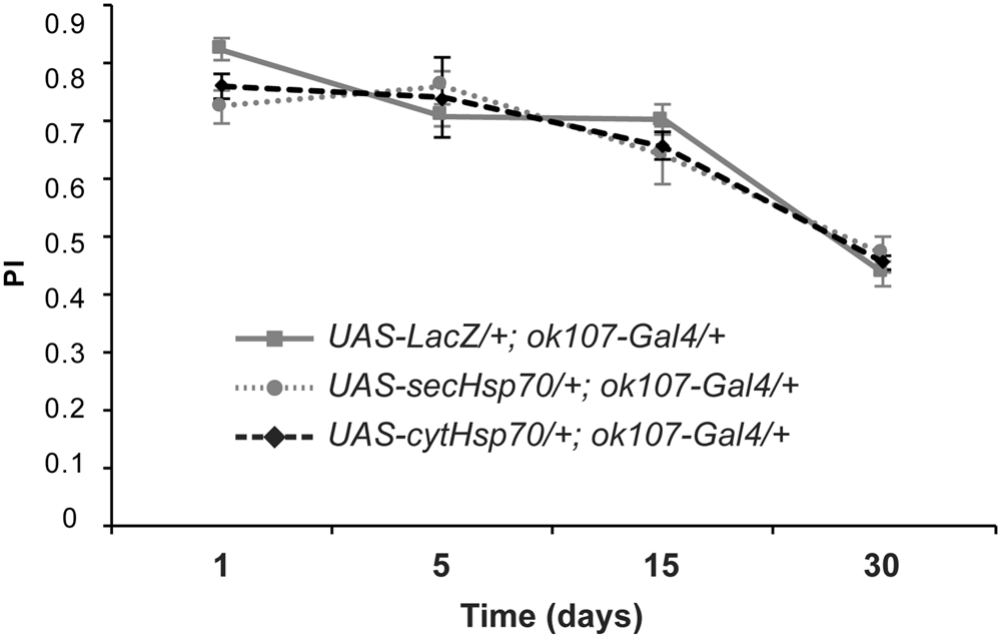
Cytosolic and secreted forms of Hsp70 do not enhance memory performance in young or elder flies. All genotypes were raised at 27 °C. Flies were trained at days 1 (**a**), 5 (**b**), 15 (**c**) or 30 (**d**) post-eclosion using olfactory classical conditioning and then tested immediately after training. Memory performance index (P.I.) is shown for control flies (*ok107-Gal4*/+*; UAS-LacZ*/+) and flies expressing either a secreted (*ok107-Gal4*/+*; UAS-secHsp70*/+) or a cytosolic (*ok107-Gal4*/+*; UAS-cytHsp70*/+*)* form of the Hsp70 chaperone. Memory performance of flies expressing the secreted (*ok107-Gal4*/+; *UAS-secHsp70*/+; day 1, p = 0.0550; day 5, p = 0.2960; day 15, p = 0.4265; day 30, p = 0.5554) or the cytosolic (*ok107-Gal4*/+*; UAS-cytHsp70*/+; day 1, p = 0.1339; day 5, p = 0.7947; day 15, p = 0.6182; day 30, p = 0.8622) form of Hsp70 is statistically undistinguishable from the performance of control flies (*ok107-Gal4*/+; *UAS-LacZ*/+) at all ages tested. Error bars indicate SEM; n = 14 per group; Tukey’s comparison test: no significant.

Hsp70 did exhibit full protection against the memory impairments induced by Aβ42 neurotoxicity. This protective effect was observed for both isoforms, the Hsp70 delivered to the extracellular matrix as well as the one delivered to the cytosol and, in some cases, memory performance of flies expressing the Hsp70 tended to be higher than that of control flies. Remarkably, this tendency was observed in a background of upregulated Aβ42, accumulating high loads of Aβ42 (Fig. 3e). For this reason, we decided to test the potential of Hsp70 to modulate memory formation in the absence of Aβ42. Performance of flies expressing the secHsp70 (*UAS-secHsp70*/+*; ok107-Gal4*/+) was equivalent to that of control flies (*UAS-LacZ*/+*; ok107-Gal4*/+) at day 1, 5, 15 and 30 post-eclosion (Fig. 5). Interestingly, Thirty-day-old flies did not show any sign of memory improvement when compare with their respective control group non-expressing secHsp70. These results indicated that, although secHsp70 protected against the memory deficits induced by Aβ42, it did not prevent the memory decline associated with aging. Similarly, performance of flies expressing the cytosolic form of Hsp70 (*UAS-cytHsp70*/+*; ok107-Gal4*/+) was also equivalent to that of control flies (*UAS-LacZ*/+*; ok107-Gal4*/+) at day 1, 5, 15 and 30 post-eclosion (Fig. 5). Again, the intracellular function of Hsp70 did not halt the progression of memory loss related to aging. Therefore, the holdase activity of Hsp70 either in the extracellular matrix or in the cytosol specifically prevented the development of memory deficits triggered by Aβ42 neurotoxicity without enhancing memory formation in young or old flies.

## Discussion

Despite the tremendous advance experienced in the understanding of the AD pathogenesis, this knowledge has been sterile in developing therapies that efficiently reduce the symptoms of the disease. Two main molecular factors are responsible for triggering the disease: the microtubule associated protein tau, which is hyperphosphorylated and accumulates intracellularly in neurofibrillary tangles (NFT); and amyloid-β peptides, which aggregate extracellularly in plaques. Oligomers and protofibrils of Aβ42 have been proven to be the most toxic species^10^. Here we show that the onset and the degree of the cognitive impairments associated with AD are proportional to the cumulative expression level of Aβ42; the higher the level of Aβ42, the earlier the onset of the memory impairment. Remarkably, regardless of the severity observed for these memory deficits, we discovered that co-expression of the chaperone Hsp70 reverted the cognitive impairments to levels equivalent to those of control animals, supporting a potential application of this family of chaperones for palliative treatments of AD.

As Aβ42 is mostly located in the extracellular space, we engineered a form of Hsp70 that was selectively delivered to this cellular location. Both Hps70s, cytosolic and secreted, protected against the neurotoxic effects of Aβ42 expression, restoring normal levels of memory. Based on these observations, it is possible to be facing two independent mechanisms of action for Hsp70, an intracellular clearance pathway^6^ and an extracellular masking effect^24^. On the one hand, Hsp70 exhibits a variety of functions in the extracellular space. STI1 is a co-chaperone of the Hsp70/Hsp90 machinery that is located in extracellular regions and triggers cytokine-like activity^3^. This machinery is responsible for activating stress responses. In fact, Hsp70 activates microglia, and increases expression of the *insulin degrading enzyme* (*ide*), which degrades β-amyloids, and the *tumor growth factor-β1* (*tgf-β1*) in astrocytes^6^. Upregulation of *ide* and *tgf-β1* by Hsp70 has been shown to prevent the memory impairments associated with expression of APPsw in a mice model of AD and, as extracellular Hsp70 induces expression of *tgf-β1*^7^, Hsp70 has been proposed to trigger this protective effect from the extracellular space^6^. Additionally, we have previously reported that the secreted form of Hsp70, genetically engineered to be delivered to the extracellular space, protects against neurodegeneration and cell death in a *Drosophila* model of Aβ42 neurotoxicity^24^. Given the (1) highly oxidizing environment and (2) low concentrations of ATP in the extracellular space^4^, and (3) the fact that the ATPase domain of the protein (foldase activity) was dispensable for these protective effects, we proposed that the activity of Hsp70 protecting against Aβ42 neurotoxicity was meditated by its holdase activity through direct binding to Aβ42^24^. This hypothesis suggests that Hsp70 binds Aβ42 and masks the neurotoxic epitopes in its assemblies, without involving the refolding activity of Hsp70 or degradation pathways. This activity then stabilizes small Aβ42 assemblies into nontoxic complexes, masking Aβ42 neurotoxicity and preventing its interaction with cellular substrates, which inhibits the ability of Aβ42 to disrupt membranes, penetrate cells, and spread to neighboring cells^1,2,11^.

On the other hand, although Aβ42 is mainly secreted to the extracellular space, it can also be internalized by re-uptake^11^ and endocytosis^12^. Intracellular Aβ42 is highly toxic to synapses^3,28–30^, which lead to synapse loss and eventual memory deficits. Therefore, cytosolic Hsp70 could also mediate its protective effect on memory loss through its holdase activity, a process that we previously postulated under the masking hypothesis^24^. This process involves Hsp70 binding to Aβ42 oligomers and preventing their deleterious effects^24^. The masking activity (holdase activity) of Hsp70 is independent of the foldase activity characteristic of folding/re-folding functions of the protein; however, under high concentrations of ATP as those in the cytosol, Hsp70 could also participate in the re-folding of misfolded and/or aggregated proteins, including Aβ42 and hyperphosphorylated tau. Following the amyloid cascade hypothesis, the expression of Aβ42 in the present model of AD could lead to hyperphosphorylation of tau, for which cytosolic Hsp70 could potentially prevent the formation of NFTs by binding and masking this aberrant form of tau. Hsp70 has also been involved in the regulation of macromolecular complexes and the control of protein-protein interactions^3^. Additionally, Hsp70 exerts a pleiotropic effect against cellular stress. The role of Hsp70 preventing oxidative stress, mitochondrial depolarization and cell death has been previously reported^31^. Hsp70 has also been reported to activate expression of *ide* and *tgf-β*, which has been suggested to suppress the progression of AD^6^. Interestingly, overexpression of the wild type Hsp70, which is cytosolic, promotes production of IDE in astrocytes and microglia and TGF-β1 in astrocytes and neurons^6^. Although this is expected to occur through an intracellular mechanism, it was also suggested that Hsp70 would reach the extracellular space and trigger expression of *tgf-β1*^6^.

The present data suggest that the protective effects of Hsp70 operate through two main pathways: an intracellular pleiotropic effect that triggers expression of genes protecting against cellular stress, and an extracellular function by which Hsp70 binds and sequesters Aβ42 preventing its deleterious activities. The ability to independently manipulate intracellular and extracellular Hsp70s enabled discrimination of the differential effects that each of these molecular cascades exert on the various symptoms of AD. The cytosolic form seems to protect specifically against cell death and the synaptic effects underlying the loss of memory^6^ as we also show here. The secreted form, however, showed a broader effect that prevented neurodegeneration, as well as cell death and memory loss^24^, which is also presented here. Therefore, the data indicate that the different symptoms of AD exhibit a differential sensitivity to the intracellular and extracellular pathways. Although these two mechanisms occur in separate cellular compartments, they both seem to be intimately related as Hsp70 and Aβ42 are continuously transported in and out the cell.

Together, the current findings prove the therapeutic potential of the chaperone Hsp70 against Aβ42 neurotoxicity and suggest the existence of multiple pathways leading to memory impairment in the context of AD. Further research identifying additional components of the protein quality control system will contribute to achieve a better understanding of the pathophysiology of AD and to develop more effective therapies to palliate AD symptoms. The data presented here support our *Drosophila* model of Aβ42-induced neurotoxicity as one ideally suited to investigate the synaptic mechanisms underlying the memory loss associated with AD.

### Experimental procedures

#### Fly strains and conditioning

Fly stocks were raised on standard cornmeal media at 25 °C. Flies carrying the UAS transgenes *w; UAS-Aβ42*^26^, *w; UAS-secHsp70*^24^ and *w; UAS-cytHsp70*^24^ were previously described. The Gal4 line *ok107-Gal4*^32^ was obtained from the Bloomington Stock Center at Indiana University (Bloomington, IN). Crosses between flies bearing the ok107-Gal4 driver and the different UAS lines or a combination of them were set at 25 °C for 2 days and then either transferred to 27 °C or kept at 25 °C and 70% relative humidity on a 12 h light/dark cycle for development and aging until conditioning.

#### Olfactory classical conditioning

Olfactory learning was assayed using olfactory classical conditioning procedures^33^. All behavioral experiments were performed under a dim red light at 26 °C and 80% relative humidity. Groups of 50–60 flies were transferred to small plastic tubes with a copper-grid floor to deliver the electric shock. A single cycle of training consisted of simultaneous presentation (CS+) for 60 sec along with shock pulses (1.25 sec) of 90 V (GRASS S48 Stimulator) every 5 sec, followed by the second odor presentation without associated shock (CS-) for another 60 sec. Odor presentations were separated by 30 sec of fresh air. For each N, two groups of flies of the same genotype were trained and tested simultaneously with the CS+ and CS- odors reversed. Benzaldehyde and 3-octanol were selected as the odor pairs.

After training, the animals were tested immediately in a runway in which they chose between avoiding the CS+ or the CS- odor. Performance index (PI) was calculated by subtracting the number of flies avoiding the CS- odor from the number of flies avoiding the CS+ odor, divided by the total number of flies. The final PI score was calculated as the average of the two reciprocal half experiments with reversed odors.

#### Odor and shock acuity

Flies stimulus perception was evaluated by their preference to avoid an electric shock of 90 volts or the odors, octanol and benzaldehyde, naively before conditioning. Each stimulus was presented independently to flies of the different ages and the corresponding genotype. Odor and shock avoidance were calculated by subtracting the number of flies avoiding the odor or shock from the number of flies avoiding mineral oil (the solvent for the odors), divided by the total number of flies.

#### Western blot

Proteins extracts were obtained from ten fly heads homogenized in 20 μl of RIPA buffer containing protease and phosphatase inhibitors. Samples were then incubated for 1 h at 55 °C in monomerization buffer (9 M Urea, 1% SDS, 25 mM Tris-HCl, 1mMEDTA), and followed by centrifugation at 14.000 rpm for 2 mins. Supernatants were boiled for 5 mins in NuPAGE LDS sample buffer (Invitrogen) and fractionated by SDS-PAGE in 12% Bis-Tris. Following transfer, 0.22 μm nitrocellulose membranes were boiled in PBS for 5 mins and then probed against mouse monoclonal Aβ42 (6E10, 1:1000) and β-Tubulin (1:1.000.000). After HRP-conjugated secondary antibody incubation, bands were visualized by enhanced chemiluminiscence (ECL).

#### Confocal Imaging

Brains were dissected in cold PBS, and then fixed for 30 min in cold 4% paraformaldehyde in PBST [0.5% Triton X-100 in PBS]. Fixed samples were washed and then mounted in Vectashield (Vector labs, USA) and observed on a Leica TCS-SP5 (Heidelberg, Germany) equipped with a motorized inverted microscope (Leitz DMIRE2). Serial optical sections (1024 × 1024 pixels) were taken at 2 µm intervals using a 40X oil immersion objective.

#### Statistical analyses

Statistical analyses were performed using GraphPad Prism (v5.0c). All data presented represent the mean ± the standard error of the mean (SEM). The sample size was 10 for each group unless otherwise stated. As PI values are normally distributed^33–35^, parametric one-way ANOVA test followed by Tukey’s post-hoc or t-test comparisons were used for statistical analysis. Wilcoxon test was used to analyze significance from zero.

#### Approvals/Regulations

The experiments described were approved by the Environmental Health and Safety Committee of the University of Florida. All the methods described were carried out in accordance with the relevant guidelines and regulations.

### Data Availability

All data and resources described in this paper will be available upon request.

## Electronic supplementary material

Supplementary Information
