# Supplementary material for: Engineered Hsp70 chaperones prevent Aβ42-induced memory impairments in a Drosophila model of Alzheimer’s disease
Source: Sci Rep. 2018 Jul 2;8:9915. doi: 10.1038/s41598-018-28341-w (PMC6028656; doi:10.1038/s41598-018-28341-w)
Supplement: Supplementary file 1 — Supplementary Information [file 41598_2018_28341_MOESM1_ESM.pdf]

Engineered Hsp70 chaperones prevent Aβ42-induced memory impairments in a *Drosophila* model of Alzheimer's disease

Alfonso Martín-Peña[1,2,*], Diego E. Rincón-Limas[1,3] and Pedro Fernandez-Fúnez[4*]

1-Department of Neurology, McKnight Brain Institute, University of Florida, Gainesville, FL, USA; 2-Center for Smell and Taste, University of Florida, Gainesville, FL, USA; 3-Department of Neuroscience and Center for Translational Research on Neurodegenerative Diseases, Genetics Institute, University of Florida, Gainesville, FL, USA; 4-Department of Biomedical Sciences, University of Minnesota Medical School, Duluth Campus, Duluth, MN, USA; *Co-corresponding authors

Contact:

Alfonso Martín-Peña, PhD

College of Medicine, University of Florida

1600 Archer Rd, Gainesville, FL 32611

e-mail: alfonso.m.pena@ufl.edu

Phone: 352-273-7087

and

Pedro Fernandez-Fúnez, PhD

Department of Biomedical Sciences

University of Minnesota Medical School, Duluth Campus

1035 University Drive, Duluth, MN 55812

e-mail: pfernand@d.umn.edu

Phone: 218-726-6863

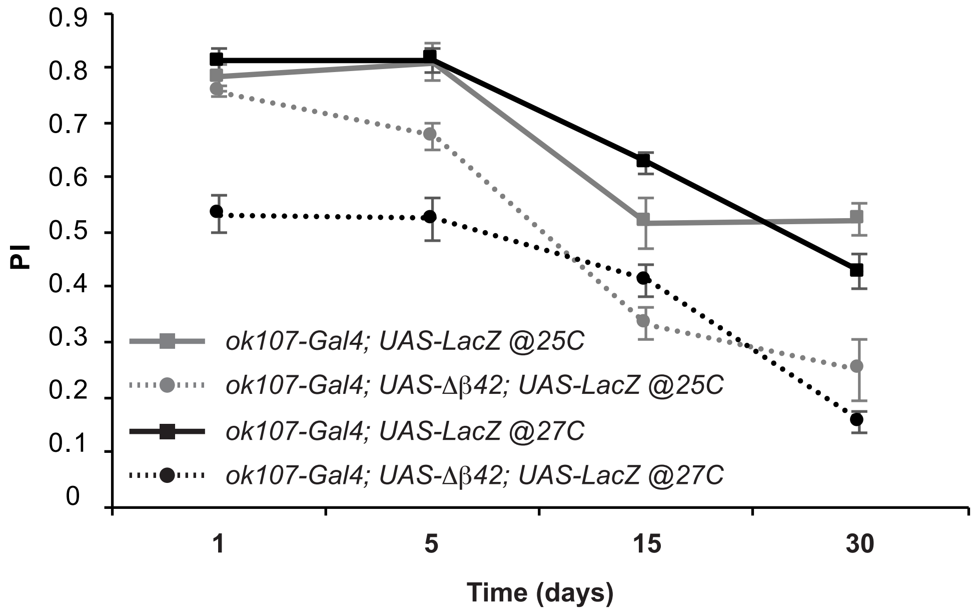

Supplementary Figure 1. Comparison of memory impairments based on the Aβ42 expression level. Flies were raised at either 25˚C or 27˚C throughout development and up to day 1, 5, 15 or 30 post-eclosion and then trained using olfactory classical conditioning. Flies were tested immediately after conditioning. Memory performance index (P.I.) is shown for control flies (*UAS-LacZ/+; ok107-Gal4/+*) and flies expressing Aβ42 in the MB neurons (*UAS-Aβ42/+; UAS-LacZ/+; ok107-Gal4/+*). At 25˚C, control flies show normal memory decay through aging and equivalent to that observed at 27˚C. Expression of the Aβ42 peptide in the MB neurons (*UAS-Aβ42/+; UAS-LacZ/+; ok107-Gal4/+*) induces a progressive memory decline at 25˚C that is comparable to that observed at 27˚C only 15 days after eclosion. Error bars indicate SEM; n=10 per group.

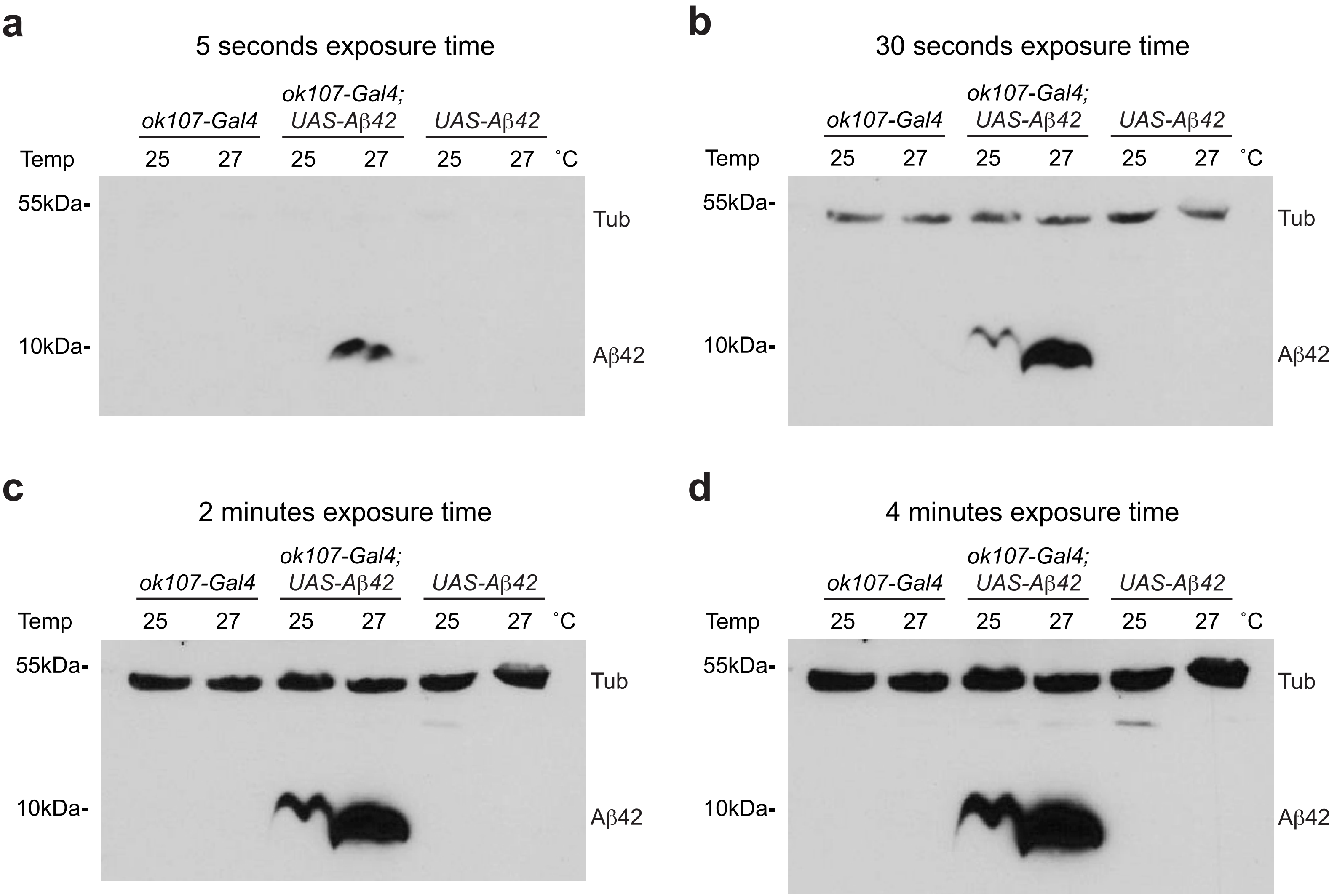

Supplementary Figure 2. Aβ42 and β-Tubulin immunodetection at day 1 post-eclosion in protein extracts from control flies (*UAS-LacZ/+; ok107-Gal4/*+ and *UAS-A*β*42/*+) and flies expressing Aβ42 in the MBs (*UAS-A*β*42/+; ok107-Gal4/*+) raised at 25˚C or 27˚C. Film from Figure 1a exposed for 5 seconds (**a**), 30 seconds (**b**), 2 minutes (**c**) and 4 minutes (**d**).

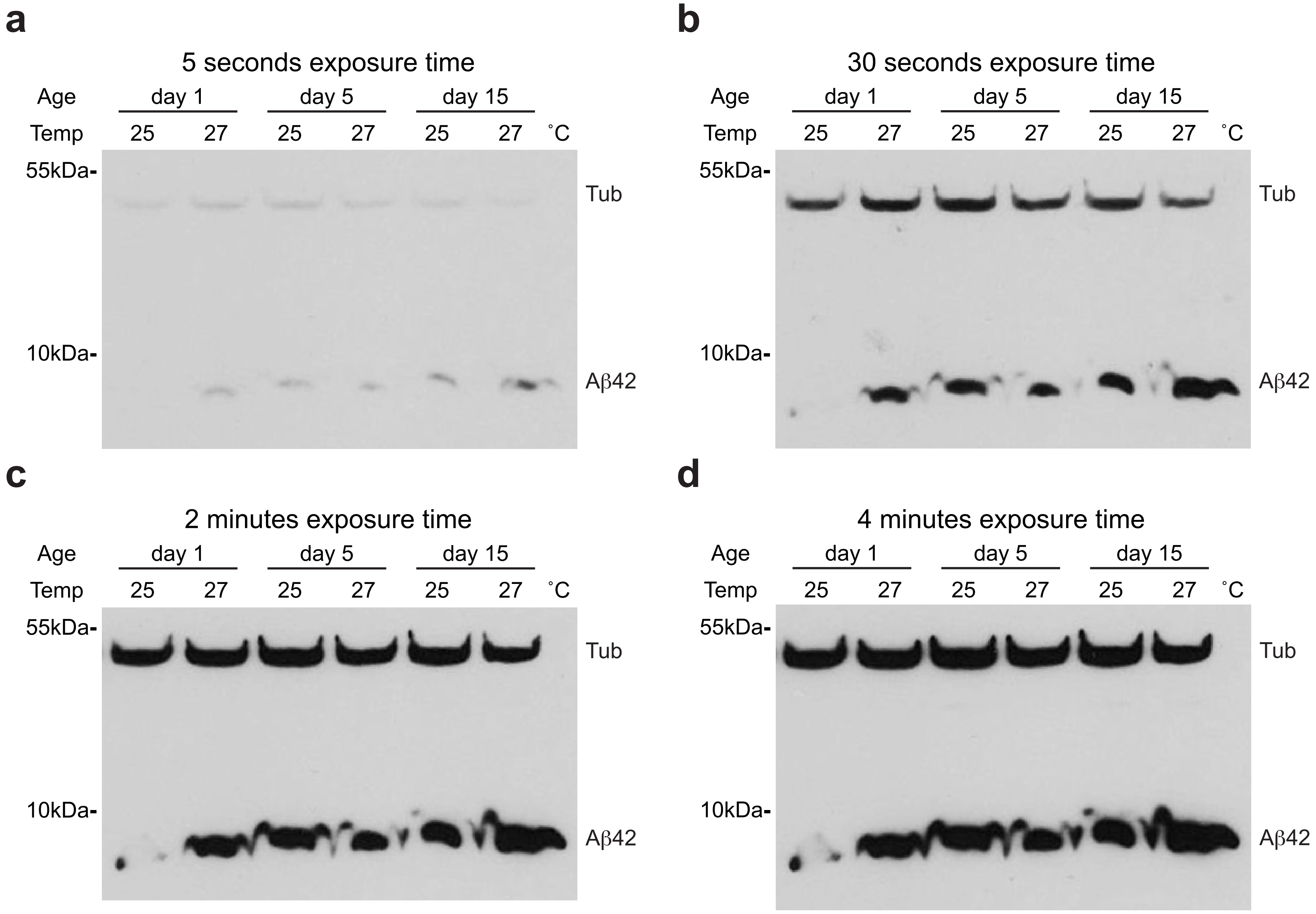

Supplementary Figure 3. Aβ42 immunodetection at day 1, 5 and 15 post-eclosion in protein extracts from flies (*UAS-Aβ42/+; ok107-Gal4/+*) raised at 25˚C or 27˚C. Film from Figure 1e exposed for 5 seconds (**a**), 30 seconds (**b**), 2 minutes (**c**) and 4 minutes (**d**).

**90V Shock Avoidance/ Age (days)**	*UAS-LacZ/+; ok107-Gal4/+*	*UAS-Aβ42/+; UAS-LacZ/+; ok107-Gal4/+*	***p-value***
**1**	0.7214 ± 0.0362	0.7342 ± 0.0389	*0.9999*
**5**	0.6744 ± 0.0512	0.7611 ± 0.0368	*0.9106*
**15**	0.6229 ± 0.0348	0.6029 ± 0.0346	*0.9999*
**30**	0.4212 ± 0.0452	0.3765 ± 0.0531	*0.9998*

Supplementary Table 1. Electric shock avoidance of flies expressing Aβ42 over time. Avoidance to an electric shock of 90 Volts for control flies (*UAS-LacZ/+; ok107-Gal4/+*) and flies expressing Aβ42 (*UAS-Aβ42/+; UAS-LacZ/+; ok107-Gal4/+*) and their t-test *p-value* for statistical significance.

**3-Octanol Avoidance/ Age (days)**	*UAS-LacZ/+; ok107-Gal4/+*	*UAS-Aβ42/+; UAS-LacZ/+; ok107-Gal4/+*	***p-value***
**1**	0.7939 ± 0.0505	0.7028 ± 0.0315	*0.8531*
**5**	0.8116 ± 0.0121	0.5096 ± 0.1088	*0.0616*
**15**	0.4048 ± 0.0710	0.3438 ± 0.0568	*0.9999*
**30**	0.3624 ± 0.0793	0.3774 ± 0.0871	*0.3698*

Supplementary Table 2. Odor avoidance for 3-octanol of flies expressing Aβ42 over time. Avoidance to the odor 3-octanol for control flies (*UAS-LacZ/+; ok107-Gal4/+*) and flies expressing Aβ42 (*UAS-Aβ42/+; UAS-LacZ/+; ok107-Gal4/+*) and their t-test *p-value* for statistical significance.

**Benzaldehyde Avoidance/ Age (days)**	*UAS-LacZ/+; ok107-Gal4/+*	*UAS-Aβ42/+; UAS-LacZ/+; ok107-Gal4/+*	***p-value***
**1**	0.4338 ± 0.0485	0.3145 ± 0.0425	*0.8395*
**5**	0.3944 ± 0.0805	0.1420 ± 0.1144	*0.5339*
**15**	0.2258 ± 0.0480	0.1723 ± 0.0343	*0.9999*
**30**	0.2759 ± 0.0932	0.0892 ± 0.0497	*0.2403*

Supplementary Table 3. Odor avoidance for benzaldehyde of flies expressing Aβ42 over time. Avoidance to the odor benzaldehyde for control flies (*UAS-LacZ/+; ok107-Gal4/+*) and flies expressing Aβ42 (*UAS-Aβ42/+; UAS-LacZ/+; ok107-Gal4/+*) and their t-test *p-value* for statistical significance.

Genotypes	1	2	3	4
**1**	-	*0.9327*	*0.9901*	*0.7899*
**2**	-	-	*0.9833*	*0.9991*
**3**	-	-	-	*0.9229*

Supplementary Table 4. Tukey's comparison test *p-values* at day 1. Table shows *p*-values for memory performance between paired genotypes at 25C. Genotypes are 1- *UAS-LacZ/+; ok107-Gal4/+*, 2- *UAS-Aβ42/+; UAS-LacZ/+; ok107-Gal4/+*, 3- *UAS-Aβ42/UAS-secHsp70/+; ok107-Gal4/+*, and 4- *UAS-Aβ42/+; UAS-cytHsp70/+; ok107-Gal4/+*.

Genotypes	1	2	3	4
**1**	-	*0.1225*	*0.4367*	*0.6424*
**2**	-	-	*0.8109*	*0.6834*
**3**	-	-	-	*0.9921*

Supplementary Table 5. Tukey's comparison test *p-values* at day 5. Table shows *p*-values for memory performance between paired genotypes at 25C. Genotypes are 1- *UAS-LacZ/+; ok107-Gal4/+*, 2- *UAS-Aβ42/+; UAS-LacZ/+; ok107-Gal4/+*, 3- *UAS-Aβ42/UAS-secHsp70/+; ok107-Gal4/+*, and 4- *UAS-Aβ42/+; UAS-cytHsp70/+; ok107-Gal4/+*.

Genotypes	1	2	3	4
**1**	-	*0.0210*	*0.8839*	*0.9373*
**2**	-	-	*0.0043*	*0.0083*
**3**	-	-	-	*0.9994*

Supplementary Table 6. Tukey's comparison test *p-values* at day 15. Table shows *p*-values for memory performance between paired genotypes at 25C. Genotypes are 1- *UAS-LacZ/+; ok107-Gal4/+*, 2- *UAS-Aβ42/+; UAS-LacZ/+; ok107-Gal4/+*, 3- *UAS-Aβ42/UAS-secHsp70/+; ok107-Gal4/+*, and 4- *UAS-Aβ42/+; UAS-cytHsp70/+; ok107-Gal4/+*.

Genotypes	1	2	3	4
**1**	-	*0.0010*	*0.8084*	*0.9784*
**2**	-	-	*0.0001*	*0.0039*
**3**	-	-	-	*0.6058*

Supplementary Table 7. Tukey's comparison test *p-values* at day 30. Table shows *p*-values for memory performance between paired genotypes at 25C. Genotypes are 1- *UAS-LacZ/+; ok107-Gal4/+*, 2- *UAS-Aβ42/+; UAS-LacZ/+; ok107-Gal4/+*, 3- *UAS-Aβ42/UAS-secHsp70/+; ok107-Gal4/+*, and 4- *UAS-Aβ42/+; UAS-cytHsp70/+; ok107-Gal4/+*.
